# The Contagiousness of the General Symptoms of Syphilis

**Published:** 1868

**Authors:** Freeman J. Bumstead

**Affiliations:** Professor of Venereal Diseases at the College of Physicians and Surgeons, New York


### ARTICLE III.

*The Contagiousness of the General Symptoms of Syphilis.* By Freeman J. Bumstead, M. D., Professor of Venereal Diseases at the College of Physicians and Surgeons, New York.

The publication in the April number of this Journal of a highly interesting article by Dr. Sigmund, entitled, "Is Tertiary Syphilis Communicable," calls forth a few comments and suggestions, which, it is hoped, may not be without interest to the readers of the Archives.

The fact that any of the general symptoms of syphilis are contagious, has only of late years been placed beyond dispute. How and why it was so long ignored is known to every one, viz., through the great weight justly attached to the name of Ricord, who, however, confined his experimental inoculations of the secretion of general symptoms either to the patients themselves, or to persons already infected with syphilis; and since syphilis, like small pox, vaccinia, etc., is a diathetic disease, his inoculations were necessarily failures.

On the other hand, Ricord formerly admitted no radical distinction between the chancroid and true chanchre, and since his inoculations proved the auto-inoculability of a large number of so-called "primary ulcers," the conclusion with him and his school was inevitable, that primary syphilis was communicable, and general syphilis not.

Alleged cases of the successful inoculation of secondary symptoms had indeed been reported by Wallace and others, but here there would appear to have been a stumbling-block in the way of the recognition of their value. In the history of all such cases, it was evident that the disease commenced in the recipient with a chancre, attended with its pleiad of indurated ganglia, and followed by the usual period of incubation preceding the outbreak of secondary symptoms. By some strange fatuity, it was thought that if a mucous patch was communicable at all, a mucous patch must be the result; that the secretion of a chancre alone could produce a chancre; and hence it was inferred that Wallace must have been mistaken regarding the source of his virus, and have taken it from a chancre, possibly in course of transformation into a mucous patch, as was known not unfrequently to occur.

The explanation of these difficulties—and it is so simple that it is a wonder it was never thought of before—was finally discovered; two diseases had been confounded under the name of syphilis, one of them local, the other constitutional; the former auto-inoculable, the latter not *auto*-inoculable, but inoculable upon, and communicable to, any person not already under the influence of the diathesis, in its secondary as well as in its primary stage. Moreover, it was found that this latter disease, from whatever stage its virus might be derived, would run its course, as any sensible disease would do, starting with its cradle (chancre), enjoying its youth (secondary), and advancing to its old age (tertiary). What disease is there, forsooth, that springs at once into full manhood?

Only twelve years have elapsed since 1856, when Langlebert first expressed the opinion, founded on but two cases, that secondary contagion would produce a chancre, and it was not until the abundant proof adduced in favor of this idea by Rollet in 1859, that the fact was generally accepted, or was even generally known. The time which has since elapsed has evidently been too short for a full investigation of the limits of the contagiousness of the general symptoms of syphilis. In most of the reported cases, the infecting lesion has belonged to the secondary period, and has usually been a mucous patch. Independently of any greater degree of virulence of the syphilitic virus in the secondary, over the tertiary period, there are two reasons why mucous patches should be a fruitful source of contagion:

First. This lesion occupies those portions of the body, the orifices of mucous canals, where contact is most likely to take place with other persons (immediate contagion), or where the virus is most likely to be collected upon common household utensils and conveyed to others (mediate contagion).

Second. This lesion is one of the most persistent and most prone to return of all those belonging to syphilis.

Thus we find mucous patches upon the nipple of its wet-nurse, and those about vulva in women a fruitful source of primary syphilis upon the penis in men. I have met with a number of instances in which young men with *plaques muqueuses* upon the lips have inoculated the lips of their sweet-hearts; also with men, who in illicit intercourse contracted a primary sore in the neighborhood of the mouth instead of the more usual situation, without any unnatural mode of indulgence.

Instances of mediate contagion from secondary lesions are also not uncommon. Take, for example, the case reported by Rollet, in which a housekeeper contracted syphilis by using a spoon shortly after it had been used by her cook, who had mucous patches on her mouth; take the extension of syphilis among the glass blowers in France, who use the same tube in common for blowing; also the repeated instances occurring in the practice of an aurist in Paris, in which this disease was conveyed to a number of patients by means of a eustachian catheter. I recently observed a case in which a young man contracted the disease through the medium of the mouth-piece to his pipe, which he lent a friend who called at his room, and which he smoked immediately afterwards. The most remarkable case I ever met with was a chancre developed upon the under surface of the upper eye-lid in a man who must have contracted the disease from a contaminated towel.

But, in addition to mucous patches, the contents of the pustules of ecthyma have been proved to be contagious (Vidal); and the virulence of the blood of syphilitic persons has been placed beyond dispute by Pellizari. Now that this latter fact is established, is there not reason to believe, that, although the syphilitic virus may be more contagious in the early than in the late stages of the disease, it does not entirely lose this power at any period of its activity?

Certainly, there is no such marked distinction between secondary and tertiary syphilis as would lead us to suppose that the former possesses a power which is entirely wanting in the latter.

The distinction, after all, between the two stages is mainly an arbitrary one, and patients often present symptoms belonging to both at the same time.

While the contagiousness of tertiary syphilis is highly probable, it is still difficult to point to cases which demonstrate it. The case reported by Dr. Sigmund, for instance, appears to me inconclusive on this point. Dr. S. states that his patient "was laboring under tertiary syphilis, and had also at that time some indolent sores on the inside of the lips." But to what stage of the disease did the sores, from which the virus was derived, belong? Were they syphilitic gummata? Probably not, because such very rarely occur in this situation. It is much more likely that they were ulcerated mucous patches, so common about the mouth, ot the secondary period, which had overlapped, so to speak, the development of tertiary symptoms on other parts of the body, as occurs so frequently, especially in cases of what may be called galloping syphilis. In the absence of further details, Dr. Sigmund's case must, therefore, be regarded as one of the communication of *secondary* and not of tertiary syphilis.

The least doubtful case of the contagiousness of tertiary syphilis that I ever heard of was one that I treated by letter a few years ago:

The patient was a well-known surgeon of the West, who was called upon to operate upon a case of extensive syphilitic necrosis of the cranium, in a patient who had had no secondary symptoms for a period of two years. One of the surgeon's fingers was abraded, and at this spot a chancre was developed a week or two afterwards, and was followed by the usual train of general symptoms. Such at least was the history communicated to me by this surgeon, who was also confident that he had not been brought in contact with a case of syphilis before for several years, and could have contracted the disease only from this operation. His age, standing, and powers of observation are such as to add great weight to his statements, and I am inclined to believe his opinion correct, yet the possible sources of error are so numerous, that I would not adduce the case as one beyond question.

Undoubted proof of the contagiousness of the late symptoms of syphilis to the point of demonstration must be sought for either in experimental inoculation of some of the few tertiary lesions which furnish a secretion necessary for the purpose, or in the clinical observation of cases in which all sources of error are absent.

Such proof, I believe, will yet be found.

